# Molecular Dynamics Simulation Study and Hybrid Pharmacophore Model Development in Human LTA4H Inhibitor Design

**DOI:** 10.1371/journal.pone.0034593

**Published:** 2012-04-05

**Authors:** Sundarapandian Thangapandian, Shalini John, Mahreen Arooj, Keun Woo Lee

**Affiliations:** Division of Applied Life Science (BK21 Program), Systems and Synthetic Agrobiotech Center (SSAC), Plant Molecular Biology and Biotechnology Research Center (PMBBRC), Research Institute of Natural Science (RINS), Gyeongsang National University (GNU), Jinju, Republic of Korea; University of Akron, United States of America

## Abstract

Human leukotriene A4 hydrolase (hLTA4H) is a bi-functional enzyme catalyzes the hydrolase and aminopeptidase functions upon the fatty acid and peptide substrates, respectively, utilizing the same but overlapping binding site. Particularly the hydrolase function of this enzyme catalyzes the rate-limiting step of the leukotriene (LT) cascade that converts the LTA4 to LTB4. This product is a potent pro-inflammatory activator of inflammatory responses and thus blocking this conversion provides a valuable means to design anti-inflammatory agents. Four structurally very similar chemical compounds with highly different inhibitory profile towards the hydrolase function of hLTA4H were selected from the literature. Molecular dynamics (MD) simulations of the complexes of hLTA4H with these inhibitors were performed and the results have provided valuable information explaining the reasons for the differences in their biological activities. Binding mode analysis revealed that the additional thiophene moiety of most active inhibitor helps the pyrrolidine moiety to interact the most important R563 and K565 residues. The hLTA4H complexes with the most active compound and substrate were utilized in the development of hybrid pharmacophore models. These developed pharmacophore models were used in screening chemical databases in order to identify lead candidates to design potent hLTA4H inhibitors. Final evaluation based on molecular docking and electronic parameters has identified three compounds of diverse chemical scaffolds as potential leads to be used in novel and potent hLTA4H inhibitor design.

## Introduction

**Figure 1:**
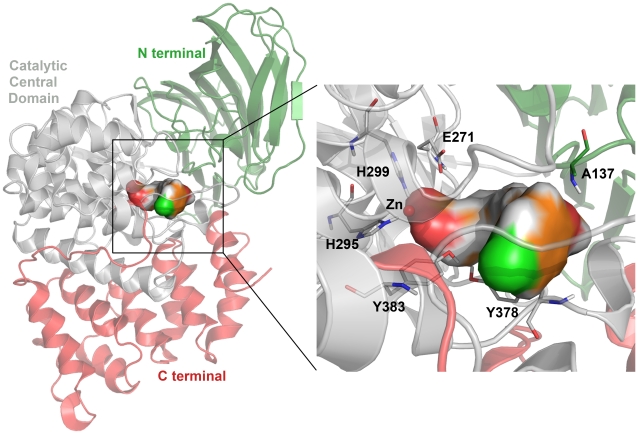
Overall 3D structure of hLTA4H enzyme (PDBcode: 3FH7) representing the three distinct domains and the zoomed view clearly shows the important catalytic residues. The bound inhibitor is shown in colored surface.

A ubiquitously present 64 kDa metal (Zn^2+^) containing cytosolic human leukotriene A4 hydrolase (hLTA4H) is a bi-functional enzyme with epoxide hydrolase and aminopeptidase activities utilizing the same Zn present active site [1]. The development and regulation of inflammation are maintained by a complex network of variety of cellular and soluble factors. These factors majorly contain eicosanoids (structurally similar paracrine hormones produced along the arachidonic acid (AA) pathway) which include the prostaglandins, the leukotrienes (LT), and the lipoxins [2]. The LT are a group of lipid mediators associated with acute and chronic inflammatory diseases particularly asthma, rhinitis, and atherosclerosis [3]–[5]. Biosynthesis of LT promotes the phosphorylation and membrane translocation of cytosolic phospholipase A2 (cPLA2) and 5-lipoxygenase (5-LO) which are the major enzymes in AA pathway. The cPLA2 releases the AA from membrane lipids followed by the action of 5-LO enzyme assisted by five-lipoxygenase activating protein (FLAP) to form the unstable epoxide LTA4. This key intermediate is subsequently converted in to LTB4 and LTC4 by the hydrolase activity of LTA4H and by glutathione transferase activity of LTC4 synthase (LTC4S) enzymes, respectively [6]. The very little known aminopeptidase activity of LTA4H has recently speculated that the enzyme may process peptides related to inflammation and host defense [7], [8]. The LTB4 is a potent pro-inflammatory activator of inflammatory responses mediated through G-protein-coupled receptors, namely, BLT1 and BLT2. The LTB4 plays an important role in amplification of many inflammatory disease states such as asthma [9], inflammatory bowel disease [10], chronic obstructive pulmonary disease [11], [12], arthritis [13], [14], psoriasis [15], and atherosclerosis [16]. It is also recently reported that increased production of LTB4 is associated with the increased risk for myocardial infarction and stroke [17]. Therefore, a therapeutic agent that inhibits the response of cells to LTB4 or the biosynthesis of LTB4 may be useful for the treatment of various inflammatory conditions. Inhibition of hLTA4H as therapeutic strategy is exemplified by the development of multiple inhibitors from different chemotypes [17]–[22]. In the development of LTA4H inhibitors over the past 15–20 years, the early approaches were based on the natural substrate followed by the utilization of already known inhibitors of zinc-containing proteins. These approaches led to the design of a number of peptide and non-peptide analogs containing zinc-chelating moieties [23]. Many 3D crystal structures of LTA4H enzyme bound with diverse inhibitors were determined and available in protein data bank (PDB). However, the substrate (LTA4) bound crystal structure has not been solved yet and that prevents the deeper insight of structural behavior of the enzyme to accommodate the long chain fatty acid. The enzyme-inhibitor crystal structure complexes provide details to understand the inhibitor binding mode and the structural changes upon inhibitor binding. The 3D structure of LTA4H enzyme is comprised of three distinctive domains, namely, C-terminal, N-terminal, and a central catalytic domain. The N-terminal domain (residues 1–207) is composed of a large seven-stranded mixed β-sheet and two smaller β-sheets whereas the C-terminal domain (residues 451–610) is formed by two layers of parallel α-helices in which the inner layer contains five and outer layer contains four arranged in anti-parallel manner. The catalytic domain that is made of residues between 208 and 450 is surprisingly sharing high structural homology to the bacterial protease thermolysin [24], . In terms of sequence identity, their similarity majorly confined to the zinc binding motif (HEXXH-X_18_-E). This catalytic domain consists of two lobes including one main α-helical and one mixed α-β lobe. The Zn^2+^ site is present between these lobes and the residues H295, H299, and E318 from these lobes co-ordinate with the metal ion (Figure 1). During the binding of substrate or inhibitor, the epoxide group or other groups possibly form co-ordinate bonds with this metal ion [25]. Though the Zn^2+^ binding site is formed by residues from the catalytic domain the substrate and inhibitor bind the whole stretch of the active site pocket, which is 6–7 Å wide and 15 Å deep hydrophobic cavity present at the interface of all three domains [25]. From the X-ray crystal structures of LTA4H enzyme, a positively charged site was formed by R563 and K565 residues was identified in the active site cavity located at the interface of all three domains. These positively charged residues present in the first turn of an α-helix pointing towards the active site. In the proposed binding mode of LTA4, the substrate, these charged residues formed electrostatic interactions with the carboxylate group of LTA4. These direct electrostatic interactions were also observed in the X-ray crystal structures in complex with inhibitors possessing carboxylate group or similar chemotypes [26], [27]. The role of these residues as putative carboxylate recognition sites in LTA4H were reported by a site-directed mutagenesis combined with x-ray crystallography and inhibition studies. Among these residues, R563 is required to position the substrate along the catalytic elements of the active site and thereby facilitates the epoxide hydrolase activity of the enzyme [28]. The mutagenetic replacements of R563 completely abolished the epoxide hydrolase activity of LTA4H indicated the importance of this residue whereas the mutation of K565 has shown variable results. Another report showed that the mutagenetic replacements of E271 abrogate both catalytic activities (hydrolase and aminopeptidase) of the enzyme [29]. A previous study has shown the considerable differences in terms of LTA4H inhibitory activity of very similar chemical compounds [17]. We focused on these results and performed molecular dynamics (MD) simulations of LTA4H and these inhibitor complexes as the results can be used to understand about the essential molecular components to bind the active site of the enzyme with high affinity. The enzyme-substrate and -inhibitor complexes were prepared using molecular docking when their crystal structures are not available. Interesting results were observed during the investigation of molecular trajectories obtained from the MD simulations. The binding modes, structural changes, intermolecular hydrogen bonds, and interaction energy calculations have revealed the molecular interactions explaining the differences in the biological activities of the inhibitors. Two hybrid pharmacophore models were developed using MD refined enzyme-substrate and -inhibitor complexes. The insights acquired from the present study may be useful in designing novel LTA4H inhibitors as potent anti-inflammatory agents.

## Materials and Methods

### Selection of inhibitors and enzyme-inhibitor complex models

**Figure 2:**
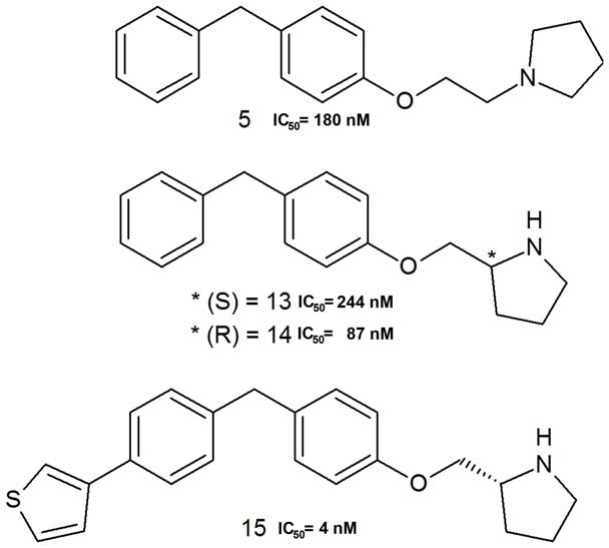
The 2D chemical structures of hLTA4H inhibitors used in this study along with their IC_50_ values.

Four inhibitors, namely, C5, C13, C14, and C15 of similar chemical structures but with highly varying LTA4H inhibitory profiles were identified from the literature (Figure 2) [17]. The LTA4H-inhibitor complexes were prepared for the MD simulations to investigate the structural details to address the observed differences in inhibitory activity profiles. The crystal structures of LTA4H complexed with C5 and C14 (PDB codes 3FH8 and 3FH5, respectively) were already available in PDB and these structures were directly used in MD simulations. As the crystal structures of C13 and C15 are not solved yet, crystal structures of LTA4H bound with inhibitory molecules similar to C13 and C15 were utilized in preparing their complexes [17], [30]. All the crystal structures were checked for their correctness with regard to their full length, added fragments, and structure of the bound inhibitors. All the selected crystal structures were missing four N-terminal amino acids in their structures and these missing regions were built using Accelrys Discovery Studio 2.5 (DS) before using them in MD simulations. The C13 is the ‘S’ isomer of C14 and therefore the bound conformation of C14 in 3FH5 was modified to represent C13 by changing its stereochemistry. In case of the most active compound C15, the very similar compound present in 3FUN was modified by removing the carbonyl oxygen atom present between the two phenyl groups. Along with these four LTA4H-inhibitor complexes LTA4H-substrate (LTA4) complex system was also prepared using 3FUN to be used in this study to compare with the structural details of inhibitor complexes. The chemical structure of LTA4 was sketched and docked into the active site of the average structure prepared from 5 ns MD simulation of LTA4H apoform.

### Molecular dynamics simulations

Initial coordinates for the protein atoms were taken from the complex structures of LTA4H-inhibitor, substrate complexes, and apoform. The protonation states of all ionizable residues were set to their normal states at pH 7. Six MD simulations were performed for systems including apoform, inhibitors and substrate complexes. All MD simulations were performed with GROMOS96 forcefield using GROMACS 4.5.3 package running on a high performance linux cluster computer [31], [32]. During the MD simulations, all the protein atoms including divalent metal ion (Zn^2+^) were surrounded by a cubic water box of SPC3 water molecules that extended 10 Å from the protein and periodic boundary conditions were applied in all directions. The systems were neutralized with Na^+^ and Cl^−^ counter ions replacing the water molecules and energy minimization was performed using steepest descent algorithm for 10,000 steps. A 100 ps position restrained MD simulations were performed for every system followed by 5 ns production MD simulations with a time step of 2 fs at constant pressure (1 atm) and temperature (300 K). The electrostatic interactions were calculated by the PME algorithm and all bonds were constrained using LINCS algorithm. A twin range cutoff was used for long-range interactions including 9 Å for van der Waals and 14 Å for electrostatic interactions. The snapshots were collected at every 1 ps and stored for further analyses of MD simulations. The system stability and behavior of the catalytic structural components present in every system were analyzed using the tools available with GROMACS 4.5.3 and PyMol programs.

### Generation of hybrid pharmacophore models

Representative structures collected from the MD simulations of C15 and LTA4 complexes were utilized in the generation of hybrid 3D pharmacophore hypotheses based on the features identified from C15 the most active compound and LTA4 the substrate. The *Feature Mapping* protocol as available in DS was used in identifying the pharmacophoric features of C15 and LTA4. An investigation based on the importance of all generated pharmacophoric features was performed to select the features to complement the very important interaction points at the active site. Utilization of this hybrid pharmacophore model development methodology is completely new and different methodology from the common feature and structure-activity relationship based pharmacophore models employed in our previous study in designing hLTA4H inhibitors [33].

### Druglike database screening and molecular docking

A druglike database was developed from the commercially available Maybridge database containing 59632 chemically diverse compounds using the following procedure [34]. However, this database is found to have number of nondruglike compounds. As it is meaningless to dock all the compounds of this database into the active site of protein target and then reject them in the later stage for their nondruglike properties, the compounds not satisfying druglike properties were excluded from the database. In order to achieve this step, compounds in the Maybridge database were subjected to various rigorous druglike filters such as Lipinski's rule of five and ADMET (absorption, distribution, metabolism, excretion, and toxicity) properties [35]. Finally 4966 compounds were selected to be in the druglike database. The *Prepare Ligands* and *ADMET Descriptors* protocol as available in DS program were used in this step. This druglike database was used in database screening to select the compounds containing the hybrid pharmacophoric features identified from both the inhibitor and substrate. This virtual screening was conducted to find novel and diverse virtual leads suitable for further development. Database searching offers the advantage that the retrieved compounds are usually more easily available for testing than those based on *de novo* design methods [36].

The druglike hit compounds that were identified to have hybrid pharmacophoric features were subjected to molecular docking studies. The *Prepare Ligands* protocol as implemented in DS was employed to change the ionization states as well as to generate different tautomers and isomers of the hit compounds. The *GOLD* program from Cambridge Crystallographic Data Centre, UK uses a genetic algorithm to dock the small molecules into the protein active site. The *GOLD* allows for a full range of flexibility of the ligands and partial flexibility of the protein. Protein coordinates from the representative structure of LTA4H-C15 complex obtained from MD simulation were used to define the active site. The active site was defined with a 10 Å radius around the bound inhibitor. The ten top-scoring conformations of every ligand were saved at the end of the calculation. Early termination option was used to skip the genetic optimization calculation when any five conformations of a particular compound were predicted within an RMS deviation value of 1.5 Å. The *GOLD* fitness score is calculated from the contributions of hydrogen bond and van der Waals interactions between the protein and ligand, intramolecular hydrogen bonds, and strains of the ligand [37]. Protein-ligand interactions were analyzed using DS and *Molegro Molecule Viewer* [38]. As a further validation, electronic parameters and binding free energies were calculated for all the known active compounds and database hits using DMol3 and AutoDock 4.2 programs [39]–[41]. The final hit compounds with superior electronic parameters and binding energies were selected.

## Results and Discussion

### Selected LTA4H inhibitors

The four LTA4H inhibitors used in this study are very similar in terms of their chemical structures but their hLTA4H inhibitory profiles (IC_50_ values) tested using the same biological assay were highly deviating with one another (Figure 2). The least active C13 is the S isomer of C14 but the observed IC_50_ value reduced three folds for C13 compared to C14. The C5 also displays the same scaffold as other compounds in the study but its IC_50_ value was 180 nM which is two folds lower than its counterpart C14 (87 nM). In the other hand, addition of thiophene ring (C15) has increased the IC_50_ value of C14 to 21 folds. This high change in the hLTA4H inhibitory profile of these similar chemical compounds has increased our interest in studying the structural reasons explaining this behavior. The identification of structural responses to these similar inhibitors with different inhibitory profiles can be used in designing highly potent compounds for the inhibition of LTA4H enzyme as novel anti-inflammatory therapeutics.

### Overall stability of the systems

**Figure 3:**
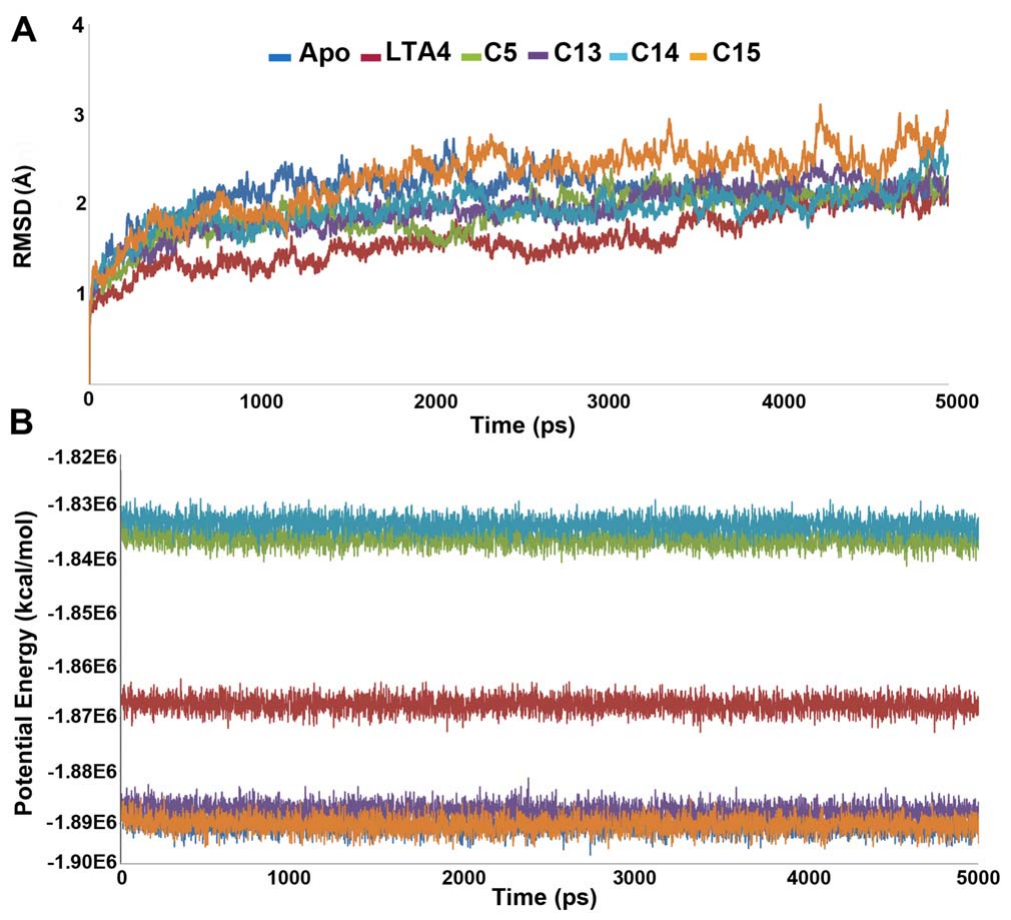
The plots to investigate the stability of the systems. (A) RMSD and (B) potential energy plots.

**Figure 4:**
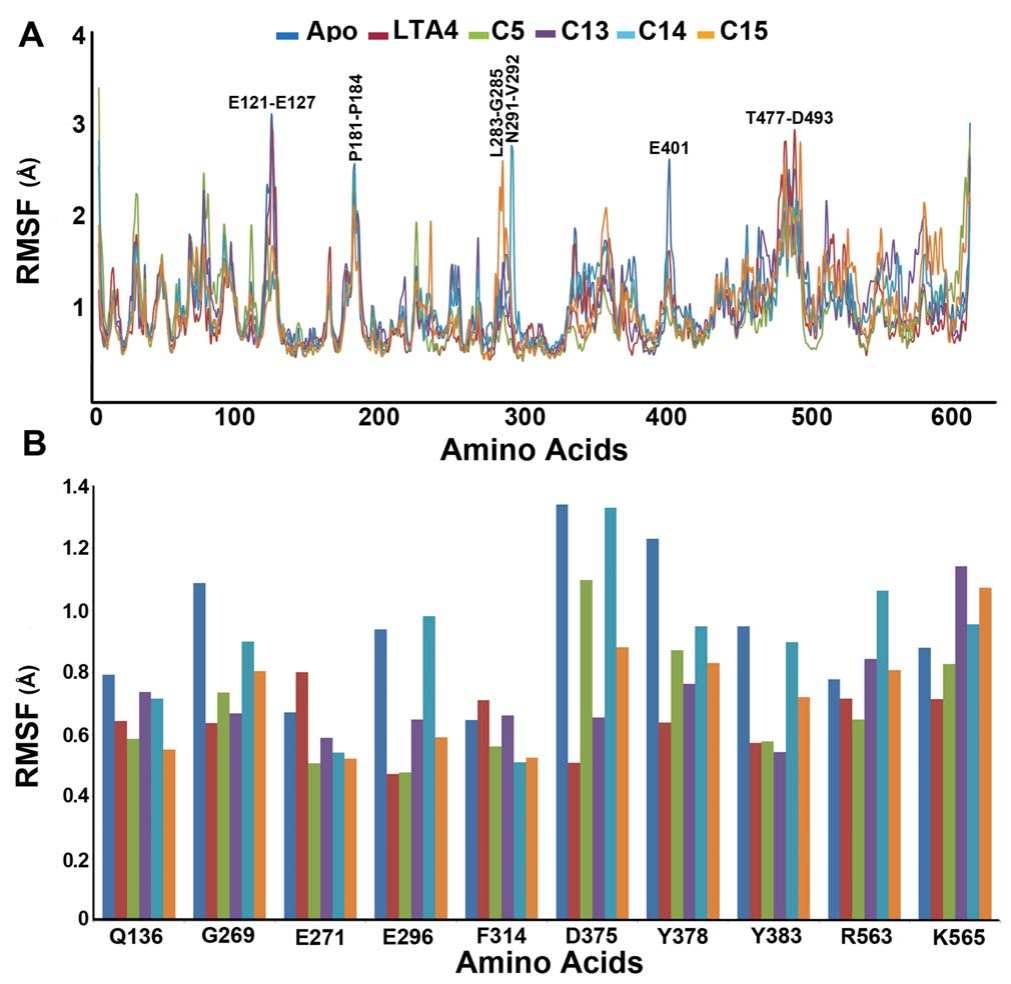
The RMSF plots of all systems. (A) full protein and (B) important active site residues.

The MD simulation results of all the systems were used in these analyses investigating the stability of every system. The stability of each protein complex during the MD simulation was also monitored using root mean square deviation (RMSD) calculation with respect to their initial structure (Figure 3A). The RMSD of LTA4H-substrate complex has shown the lowest value compared to all other systems whereas the complex with most active compound has displayed the highest among other inhibitors. Though the differences were observed in RMSD values among the systems, they all have converged at around 2.0 Å except apoform and C15 complex which had a mean value slightly over 2.0 Å. The higher RMSD value of the C15 complex (2.4 Å) depicts the structural flexibility of the system upon binding of C15 when compared to others. Comparing the mean RMSD values of C14 and C15, which are 2.0 Å and 2.4 Å, respectively, has displayed the flexibility observed in the structure because of the addition of thiophene ring in the structure. The calculated mean RMSD values of R and S isomers (C14 and C13) were 2.01 Å and 2.03 Å, respectively, showing similar stability in these complexes. Interestingly, all complexes other than the most active compound have shown the RMSD values lesser than that of apoform explaining the difference in the instability in LTA4H-C15 complex. The potential energy of the system is also a simple measure of its stability and thus plots of potential energy as a function of time were generated to observe the stability of the systems (Figure 3B). The plots indicated that all the systems in the study were well equilibrated and remained stable throughout the simulations. The apoform (−1890360 kcal/mol), C13 (−1887727 kcal/mol), and C15 (−1890238 kcal/mol) complex systems have shown lower potential energy values compared to substrate (−1867486 kcal/mol), C5 (−1836142 kcal/mol), and C14 (−1833350 kcal/mol) complexes. The root mean square fluctuation (RMSF) values of the systems were also calculated and plotted to compare the flexibility of each amino acid residues of the complex. This RMSF plot has shown the flexible regions of the systems but the focused RMSF plot has clearly displayed that none of the important catalytic residues present in the active site has shown an RMSF value more than 1.4 Å (Figure 4). This result has confirmed that the catalytic machinery present in the active site was not distorted upon binding of substrate and any of the inhibitors (Figure 4A). The amino acids, namely, G269, E296, D375, Y378, and Y383 have shown the flexibility upon the binding of different ligands. Most of these residues were highly fluctuating in apoform of the enzyme whereas they were stable in complex systems indicating the binding nature of the ligands (Figure 4B). In case of C13, C14, and C15 complexes, R563 and K565 residues were fluctuating compared to other systems. Particularly, D375 fluctuated more compared to other amino acids in the active site indicating its flexible nature in apoform and inhibitor complexes but not in substrate complex.

### Mode of substrate binding in LTA4H

**Figure 5:**
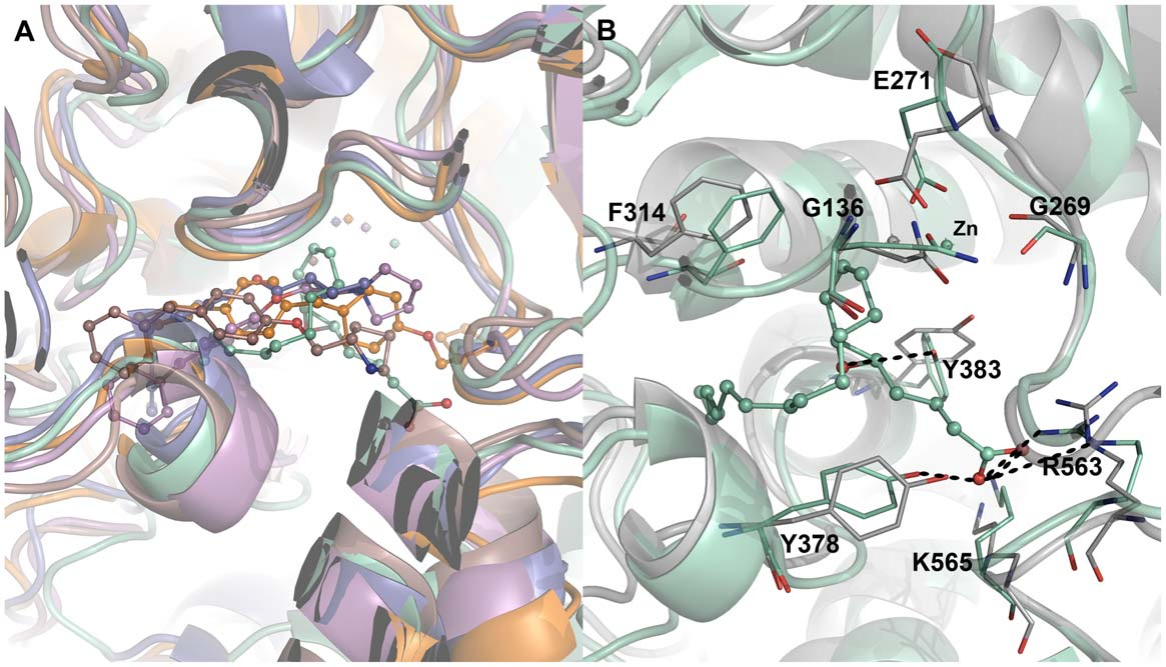
Binding modes of ligands. (A) Overlay of the binding modes of all the inhibitors at the active site of hLTA4H enzyme. The LTA4, C5, C13, C14, and C15 complexes were represented in green cyan, violet, dark salmon, slate, and orange colors, respectively. (B) Overlay of the active sites of apoform and substrate (LTA4) complex of the enzyme. Amino acid residues and bound substrate are shown in stick and ball-stick forms, respectively. The hydrogen bonds are shown in black dashed lines. The gray and green cyan cartoons represent apoform and LTA4 complexes, respectively.

**Figure 6:**
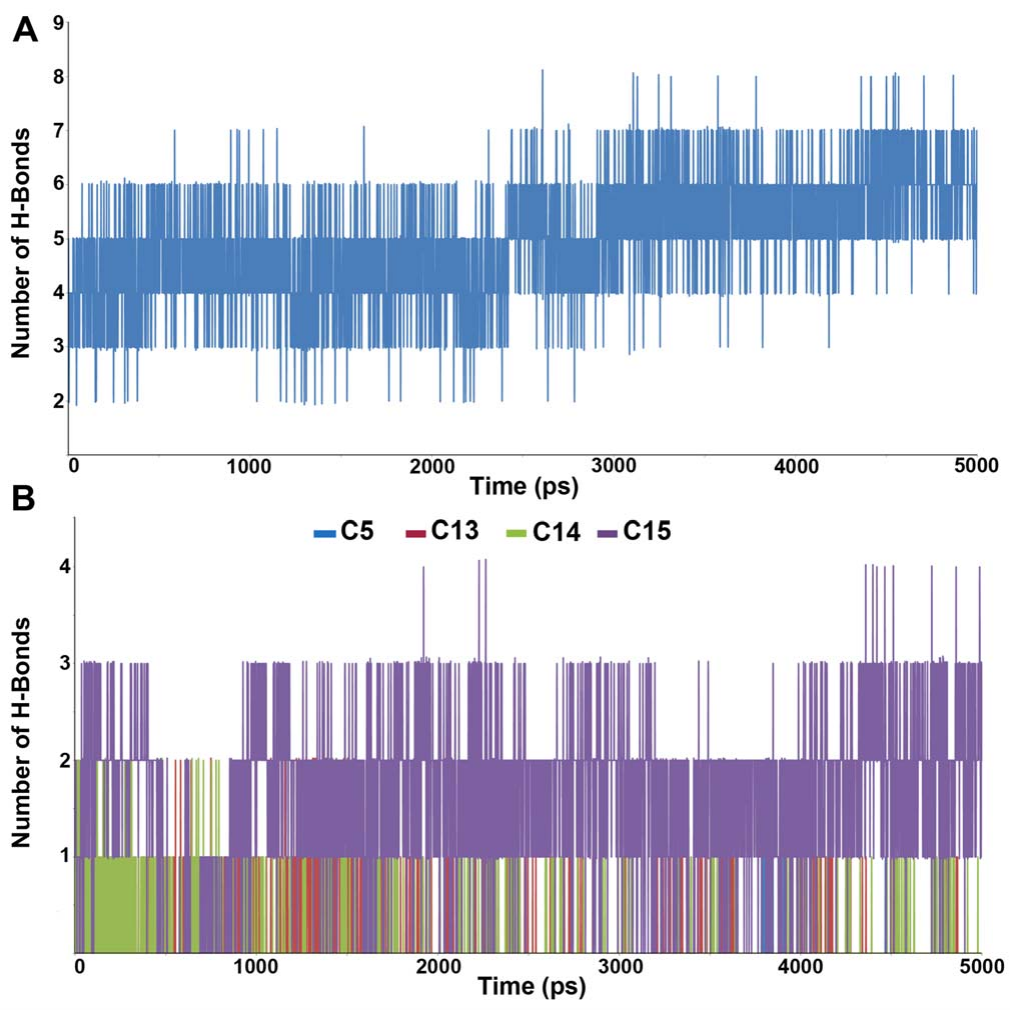
Intermolecular hydrogen bond plots. Observed intermolecular hydrogen bonds between (A) the substrate (B) inhibitors and active site residues.

The substrate LTA4 was docked into the active site of LTA4H enzyme selected as a representative structure from the MD simulation of the apoform of the enzyme. Since the crystal structure of the enzyme-substrate complex has not been determined yet, the proposed binding mode of the substrate is of great interest to understand the hydrolase mechanism of the enzyme. Some previous studies have also proposed its binding mode at the active site of the enzyme. The active site pocket lined by the amino acids belong to K21 peptide is around 6–7 Å wide and stretches 15 Å deeper into the protein [25]. The K21 is a 21 amino acids long peptide segment made of residues between L365 and K385. All the ligands under study bind the same active site pocket (Figure 5A) but they vary in terms of their molecular interactions. Particularly the calculated intermolecular hydrogen bonds have correlated well with the biological activities of the compounds under study (Figure 6). The proposed binding mode of LTA4 is observed to have its epoxide moiety close enough to the Zn^2+^ ion whereas the carboxylate group interacts with the positively charged R563 and K565 residues enabling the catalysis. The other residues such as Y378 and Y383 also have formed hydrogen bonds with the substrate (Figure 5B). The intermolecular hydrogen bonds formed in the enzyme-substrate complex were increased during the second half of the simulation time indicating the stronger interactions at the active site (Figure 6A). The long chain alkyl end of LTA4 is bent and snugly fit into the deeper hydrophobic cavity formed by residues including W311, F314 and Y378 as reported earlier [25]. Comparing to the active site of apoform, the catalytic residues in substrate complex were much stable except small side chain movements that brought the charged residues E271, R563, and K565 and the hydrophobic residues F314, Y378, and Y383 residues near the substrate for stronger molecular interactions. The lower mean RMSD values of the substrate complex (1.7 Å) also displayed the stable nature of the system compared to that of apoform (2.2 Å). These binding characteristics observed from the LTA4H-substrate simulation are compared with the binding modes of inhibitors.

### Binding characteristics of LTA4H-inhibitor complexes

#### LTA4H-C5 complex

**Figure 7:**
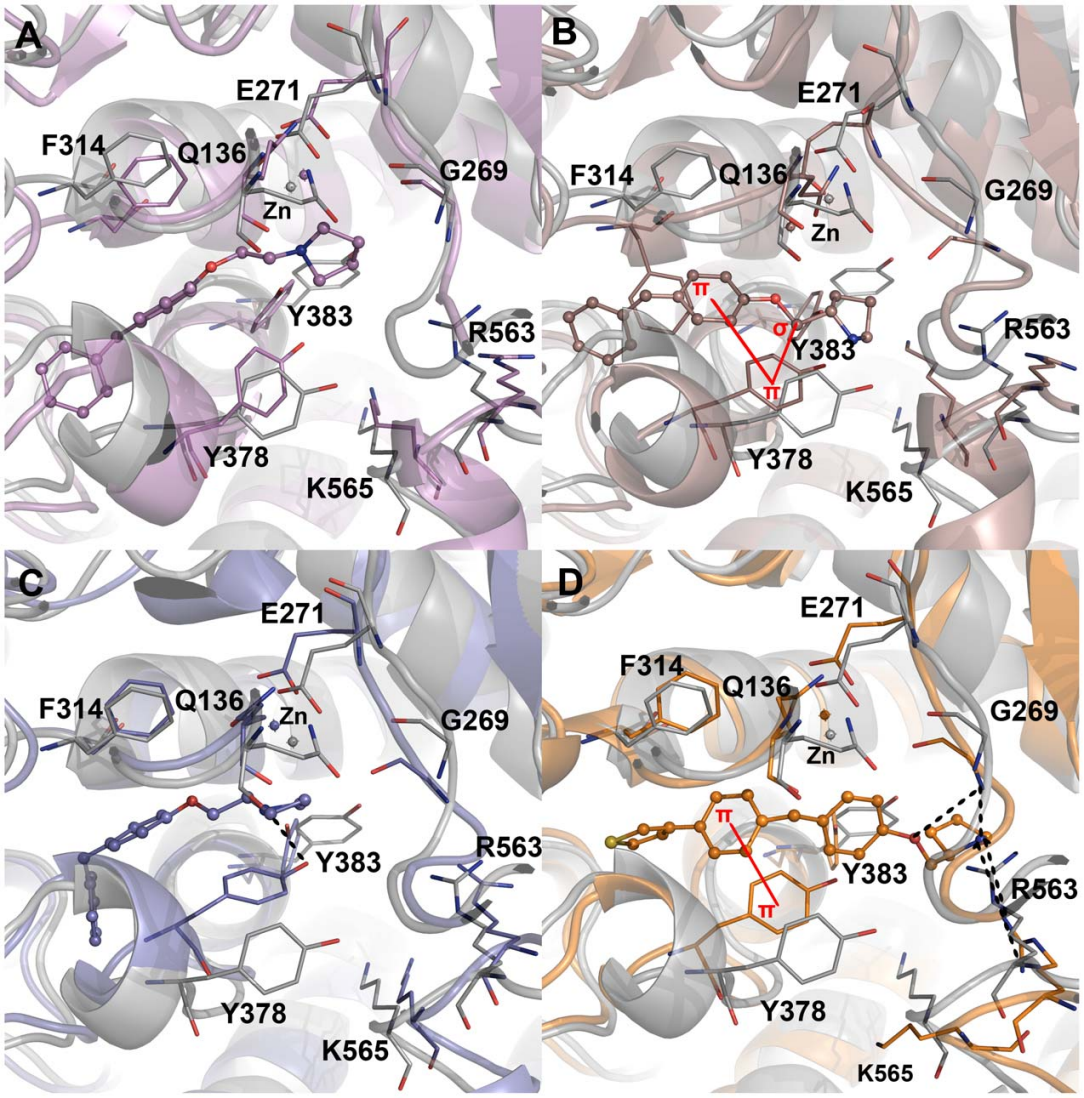
Binding modes and molecular interactions of the four inhibitors. (A) C5 in violet (B) C13 in dark salmon (C) C14 in slate (D) C15 in orange colors at the active site of hLTA4H enzyme. The hydrogen bond and π-interactions were displayed in black dashed and red solid lines, respectively. Amino acid residues and inhibitors are shown in stick and ball-stick forms whereas the gray, violet, dark salmon, slate, and orange cartoons represent C5, C13, C14, and C15 complexes, respectively.

This is the second least active compound among the inhibitors under study containing an additional methylene group and directly attached to the nitrogen atom of the five-membered pyrrolidine ring. The C13 and C14 (S and R isomers) were attached to this pyrrolidine at its second position whereas C15 differs only with an additional thiophene ring to its terminal phenyl group compared to C14. The binding mode of C5 at the active site has not formed any hydrogen bond with the active site residues throughout the simulation time indicating its binding with less affinity (Figure 6B). The region of the active site where the long alkyl chain of the substrate binds was occupied by both the phenyl rings of C5. But the compound did not stretch till the region where R563 and K565 residues are present. This unoccupied region including G269 loop has fluctuated freely during the simulation (Figure 7A).

#### LTA4H-C13 complex

The C13 is a shorter compound compared to C5 because of the additional methylene group in C5. The binding mode of this compound is also similar to C5 but the orientation of the central phenyl ring and the position of the methylene group next to the ether oxygen atom have formed two strong hydrophobic interactions with the aromatic phenol ring of Y378 such as π-π and π-σ interactions (Figure 7B). Though these hydrophobic interactions were observed in C13 complex, one or no hydrogen bonds were formed between protein and ligand molecules throughout the simulation time (Figure 6B). This is because of the binding mode that keeps the whole compound in the hydrophobic region of the active site where the long alkyl chain of the substrate binds and did not form any interactions with the residues from the carboxylate binding region. In terms of active site flexibility upon binding of C13, the phenyl ring of F314 has moved drastically down near the central phenyl ring of C13 enabling strong hydrophobic interactions. The residues including Q136, G269, E271, Y378, and Y383 have moved close to the bound inhibitor whereas the positively charged R563 and K565 residues have moved away from the inhibitor. The whole region of G269 loop was in high fluctuation during the simulation as there was no intermolecular contact that stabilized the movement (Figure 7B).

#### LTA4H-C14 complex

The C14, which is the R-isomer of C13, has formed hydrogen bonds with the active site residue Y383 and did not form any of the π interactions as observed in C13 complex. It is because of the orientation of the phenyl rings of the compound which is completely different to that of C13. The F314 residue that drastically moved down in C13 binding was stable upon C14 binding whereas Y378 that formed π-π interactions with C13 has moved close to the pyrrolidine ring of C14 to form strong hydrophobic interaction. The terminal phenyl ring of this compound bound deep into the active site region where long alkyl chain of the substrate binds unlike all other inhibitors. In this case, Q136 has moved close to the inhibitor within the interacting distance. This compound also has not reached till the carboxylate binding region of the enzyme where R563 and K565 residues are present. The upper region of G269 loop was also highly fluctuating during the simulation as it was not involved in any intermolecular interactions (Figure 7C).

#### LTA4H-C15 complex

The binding mode of C15, the most active compound, was of high affinity inside the active site of the enzyme as the binding includes strong hydrogen bond and hydrophobic interactions. The phenyl ring attached to the thiophene ring has formed a strong π-π interaction with Y378, the amino acid residue that moved close to the bound inhibitor during the simulation. The other phenyl ring attached to the ether oxygen atom was positioned in such a way to interact hydrophobically with Y383. Comparing to the positions in apoform the amino acids including Q136, E271, R563, and K565 have moved away to accommodate the inhibitor whereas G269, Y378, and Y383 have moved close to form strong interactions. One of the charged amino acids R563 and K565 as well as G269 have formed strong hydrogen bond interactions with the ether oxygen atom and pyrrolidine ring similar to the interactions observed in substrate binding (Figure 7D). These hydrogen bond interactions were stable throughout the simulation and support the experimentally observed potency in LTA4H inhibition (Figure 6B). The G269 loop was very stable upon C15 binding unlike all other inhibitor complexes and the number of intermolecular hydrogen bonds observed during the simulation was also high and stable compared to that of other complexes.

### Energetics of LTA4H-ligand complexes

The electrostatic and van der Waals (vdw) energy contributions to the non-bonded interaction energies of all ligands were calculated using *Calculate Interaction Energy* protocol implemented in DS. The total calculated electrostatic interaction energies were −130.36, −44.81, −21.58, −15.08, and 91.65 for LTA4, C5, C13, C14, and C15 complexes, respectively. These interaction energy values correlate well with the experimental IC_50_ values where C15, the most active compound, is with positive energy. The vdw energy of this compound is higher than all other compounds and it was not compensated with the electrostatic energy and thus indicates the destabilizing effect in LTA4H-C15 complex. All other complexes have shown a favorable vdw interaction energies except substrate complex where the positive vdw interaction energy was compensated by electrostatic interaction energy.

The focused investigation over the non-bonded interaction energy contribution by active site residues has shown that some residues displayed significant effects over ligand binding (Table 1). One of the positively charged residues R563 that interacts with the carboxylate moiety of LTA4, the substrate, has shown major contributions to the electrostatic energy in the ligand binding. This high electrostatic contribution is because of the interactions between negatively charged carboxylate group of LTA4 and positively charged R563 and K565 residues at the active site. The substrate complex has shown the highest contribution followed by the most active compound (C15). This residue has contributed with positive electrostatic energies for least active compounds. The negatively charged residue E271 has shown positive electrostatic interaction energies in all the complexes except C15 complex indicating the importance of this particular residue in contributing to the biological response. In terms of G269, which is present in the region where the carboxylate group of substrate binds in the active site, all the complexes have shown less contributions from electrostatic interaction energies. However, their vdw energies have contributed to the interaction energies except C15, which has gained a high positive charge against G269 explaining the repulsion between the residue and the inhibitor. The C14 and C15 compounds have shown favorable electrostatic interaction energies with D375 when compared to other ligands. The residue Y378 also has shown favorable electrostatic interaction energies for C14 and C15 as well as the substrate showing its significance in ligand binding. Finally, the divalent metal ion present in the active site has shown favorable interaction energies in all complexes except C15 complex. This can be explained based on the location of the oxygen atom of the epoxide group in LTA4 or ether group in inhibitors. In all complexes except C15, the oxygen atom of the compound is present close to the divalent cationic metal ion and thereby leads to the favorable electrostatic interactions whereas in C15 complex this oxygen atom is away from the metal ion leading to the positive electrostatic energy. These interaction energy investigations for the ligands under study have disclosed the information on the extent of involvement of active site residues and the part of ligands influencing the interactions at the active site.

**Table 1:** Calculated non-bonded interaction energies between the inhibitors and important active site residues.

Residue	LTA4		CPD5		CPD13		CPD14		CPD15	
	vdw	Elec	vdw	Elec	vdw	Elec	vdw	Elec	vdw	Elec
Q136	2.15	2.25	−3.67	−0.26	−1.78	−1.24	4.60	6.26	−1.38	2.21
G269	−0.30	3.82	−1.39	0.66	−0.67	0.97	−0.88	1.21	130.3	4.32
E271	−0.91	30.67	−0.49	7.82	−0.47	2.53	−0.43	3.70	−0.38	−6.39
E296	−0.03	32.47	−0.11	−2.90	−0.06	−0.91	0.00	−1.10	−0.49	−2.68
F314	−4.58	−1.78	−2.27	−1.67	−3.66	2.97	−2.30	2.13	−2.35	1.23
D375	−1.84	20.19	−0.90	0.87	−1.75	1.77	−0.85	−3.56	−2.29	−5.21
Y378	18.18	−18.05	−4.02	0.97	1.43	0.56	−2.69	−3.70	−5.51	−2.31
Y383	1.31	−3.58	−2.50	5.19	−0.19	1.45	−0.89	−0.99	−1.31	−0.24
R563	−2.47	−109.48	−0.09	2.85	−0.15	−2.40	−0.03	0.34	−1.37	−3.40
K565	3.04	−60.89	−0.16	−3.21	−0.99	−3.36	−0.02	6.79	−0.21	−2.65
Zn^2+^	−0.14	−86.01	−0.09	−7.16	−0.12	−12.11	−0.08	−15.85	−0.07	2.17

vdw – van der Waal; Elec – electrostatic.

### Structural changes at the active site

**Figure 8:**
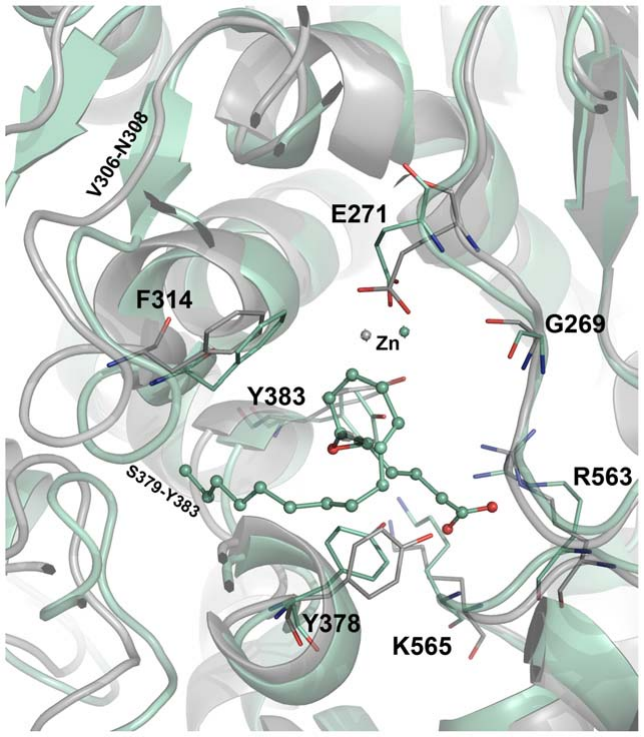
The structural changes observed between the active site regions of apoform (grey) and hLTA4H-substrate complex (green cyan). The amino acid residues are shown in thin stick form and bound substrate is shown in ball-stick form. The hydrogen atoms were hidden for clarity.

**Figure 9:**
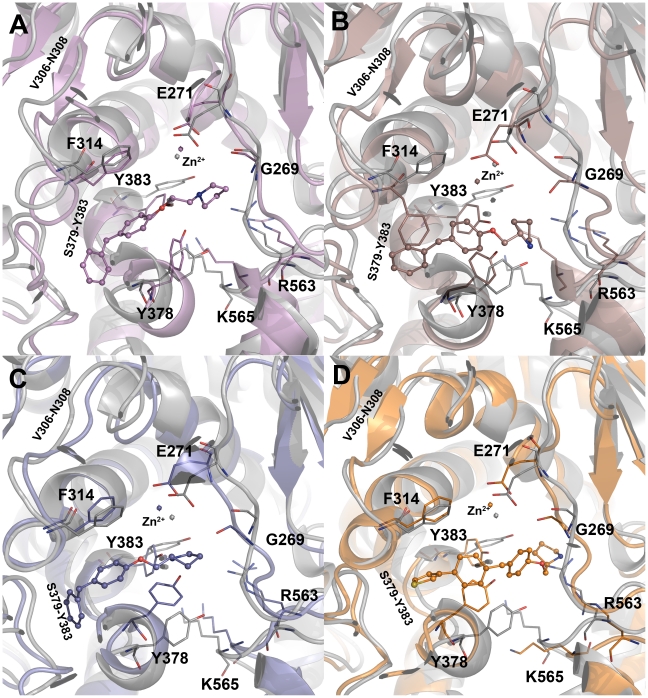
The structural changes observed at the active site region of the systems. (A) C5 (B) C13 (C) C14 and (D) C15 complexes. The amino acid residues are shown in thin stick form and bound inhibitors are shown in ball-stick form. The hydrogen atoms were hidden for clarity.

During the MD simulations, significant structural changes were observed in and around the active site influencing the way critical residues bind to the ligands. The first change observed was over the variable region formed by residues V306-N308 of the catalytic domain in apoform (Figure 8). This variable region has formed a small beta-strand upon binding of the substrate and all inhibitors except C14 (Figures 8 and 9). This change in the loop has brought significant conformational change upon the important F314 residue especially in LTA4H-C13 complex. This small beta-strand formation was not observed in the complex of C14, which is the R-isomer of C13 indicating the effect upon isomeric differences (Figure 9C). The another variable region close to the V306-N308 was also observed to form a small beta-strand but this was not significant as it was present away from the active site and has not influenced any of the active site residues in direct contact with the binding ligands. The second significant change observed in the systems is the extension of the helix formed by S379-L397 residues from the catalytic domain. In the apoform and upon substrate binding this helix remained unchanged (Figure 8) whereas upon the binding of inhibitors except C15, this long helix was extended by one to three amino acids. In case of C5 and C13 binding, this long helix was formed by the residues S379-L397 extended by three amino acids from S379-V381 whereas in C14 complex only one amino acid residue V381 involve in the formation of the long helix (Figure 9C). In C15 complex no change on the helix was observed and remained the same as observed in apoform and substrate complex (Figure 9D). These changes observed in all the systems have displayed a trend explaining the IC_50_ values of the compounds. The least active compounds C5 and C13 complexes have included three amino acid residues in the helix extension whereas the intermediate active compound C14 has extended the helix with one additional residue. In case of C15 complex, the helix has remained the same as in apoform and substrate complexes. Thus the conformational changes in this helix that has an important amino acid Y383 can influence the biological activity of the inhibitor. This amino acid residue positions its phenolic ring close to the hydrophobic phenyl rings present in the inhibitors enabling strong π-interactions at the active site.

The third significant change observed at the active site includes the very important region of the active site that reported to be involved in both the activities of the bi-functional LTA4H enzyme. This region interacts with the ligands especially the substrate through positively charged residues R563 and K565. These amino acids present at the edge of the helix formed by T567-A576 residues from the C-terminal regions. During the simulation of the systems, the conformational change over this helix was observed. The edge of this helix where K565 was present has maintained the same in apoform as well as in substrate and active compound (C14 and C15) complexes. An extension of the T567-A576 helix was observed in the least compound complexes including the K565 edge and thereby rendering the positive charged amino acid restrained and away from the inhibitor.

### Hybrid pharmacophore model generation and virtual screening

**Figure 10:**
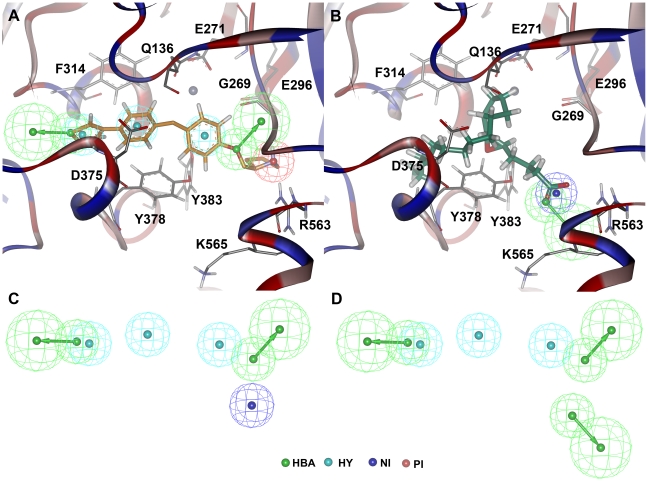
Development of hybrid pharmacophore models. The pharmacophore model generated from the binding modes of (A) C15 and (B) LTA4. The amino acid residues of the enzyme are shown in gray thin stick form whereas the bound C15 and LTA4 are shown in thick stick form. The secondary structure cartoon of the protein is colored based on the hydrophobicity of the amino acid residues. (C) Pharm-A (D) Pharm-B, the final hybrid pharmacophore models.

The representative structure obtained from the MD simulation trajectories of LTA4H-C15 complex was used in generating a pharmacophore model. The *Feature Mapping* protocol has generated all the pharmacophoric features present in the bound conformation of this most active compound at the active site of hLTA4H. The initial pharmacophore model has included overlapping ring aromatic (RA) and hydrophobic (HY) features on the phenyl and thiophene rings of the inhibitors as well as the overlapping hydrogen bond donor (HBD) and positive ionizable (PI) features over the nitrogen atom of pyrrolidine ring. The overlapping ring aromatic features were removed to simplify the pharmacophore model. Thus the final pharmacophore model included three HY, two HBA, and a PI or HBD features (Figure 10A). The PI feature generated from the nitrogen atom of pyrrolidine ring of C15 found to be repelled by the two positively charged amino acid residues R563 and K565 at the active site. These residues are very important for both the functions of LTA4H and reported to position the substrate at the active site for the effective catalysis [28]. The presence of negative ionizable (NI) or hydrogen bond acceptor (HBA) groups instead of PI or HBD groups can improve the binding of ligand by forming strong molecular interactions with these positively charged residues (Figure 10B). Hence two hybrid pharmacophore models containing all the features except PI and HBD generated from the binding mode of C15 and either a NI or HBA feature generated from LTA4 binding mode were developed (Figure 10C and 10D). Two pharmacophore models with either a terminal NI (Pharm-A) or HBA (Pharm-B) feature were developed in order to screen the chemical compounds with NI or HBA feature as present in LTA4. These final pharmacophore models were used as 3D structural queries in screening Maybridge drug-like database containing 4966 chemical compounds prepared earlier [42]. From the database screening, 7 and 226 compounds mapping all the six features of the pharmacophore model were identified by the models with NI and HBA, respectively. Finally, a total of 233 compounds satisfying all the filters were selected and subjected to the molecular docking calculations using *GOLD* program.

### Molecular docking

A total of 233 compounds identified from the database screening along with the four experimentally known compounds were docked in to the active site of hLTA4H using the representative structure of hLTA4H-C15 complex obtained from MD simulation. Interestingly the most active compound (C15) has scored a *GOLD_Fitness* value of 70.12 which is greater than other three compounds. The GOLD program was validated for its performance for hLTA4H enzyme based on the RMSD difference observed between X-ray bound and docked poses of a particular inhibitor [33]. From these docking results, 51 compounds with a *GOLD_Fitness* value greater than 70 were selected. A total of 18 compounds were selected based on the binding mode and better molecular interactions with active site components compared to that of C15 (Table 2).

**Table 2:** Results of molecular docking using GOLD and Autodock programs along with some of the electronic parameters calculated for the known inhibitors and hit compounds.

Name*	Ligand	GOLD fitness	Binding free energy kcal/mol	HOMO (eV)	LOMO (eV)	Energy gap (eV)
C15	C15	70.120	−10.86	−0.175	−0.054	0.121
C14	C14	60.090	−9.71	−0.171	−0.044	0.127
C5	C5	59.800	−9.80	−0.167	−0.034	0.133
C13	C13	62.210	−9.92	−0.180	−0.036	0.144
H1	BTB_01185	72.669	−10.34	−0.280	−0.234	0.046
H2	HTS_05216	72.383	−10.04	−0.100	−0.082	0.018
H3	GK_03083	76.419	−9.94	−0.305	−0.196	0.108
H4	BTB_11904	75.583	−9.49	−0.206	−0.144	0.062
H5	SCR_00085	72.921	−9.49	−0.192	−0.054	0.138
H6	HTS_08249	73.682	−9.31	−0.187	−0.063	0.124
H7	AW_01216	71.342	−9.15	−0.117	−0.083	0.034
H8	HTS_05096	71.885	−8.82	−0.189	−0.093	0.096
H9	SEW_01301	72.913	−8.79	−0.294	−0.224	0.070
H10	KM_10589	75.912	−8.46	−0.203	−0.077	0.127
H11	HTS_11258	73.985	−8.22	−0.279	−0.250	0.029
H12	HTS_12876	72.253	−8.03	−0.165	−0.078	0.087
H13	SPB_06794	71.083	−7.92	−0.211	−0.122	0.089
H14	HTS_12876	72.200	−7.67	−0.151	−0.086	0.065
H15	HTS_13003	70.894	−7.66	−0.254	−0.184	0.070
H16	KM_10378	70.723	−7.63	−0.177	−0.065	0.112
H17	RJC_02521	71.720	−6.50	−0.135	−0.127	0.008
H18	HTS_00131	76.041	−6.16	0.007	0.036	0.029

* H1 - Hit 1.

As an effective post-docking filtering protocol, the electronic parameters such as highest occupied molecular orbital (HOMO), least unoccupied molecular orbital (LUMO), and energy gap (ΔE) values along with the binding free energies were calculated for all compounds. The calculated energy gap values have highly correlated the experimental activities of the four compounds used in the study. A lowering trend over energy gap was observed against the experimental activity values. Thus the compounds scoring lower energy gap values than the most active compound were selected and subjected to another molecular docking procedure using Autodock program. The binding free energy values were calculated for all the compounds and three hit compounds scoring a better binding free energy values than the inhibitor compounds were selected (Table 2).

**Figure 11:**
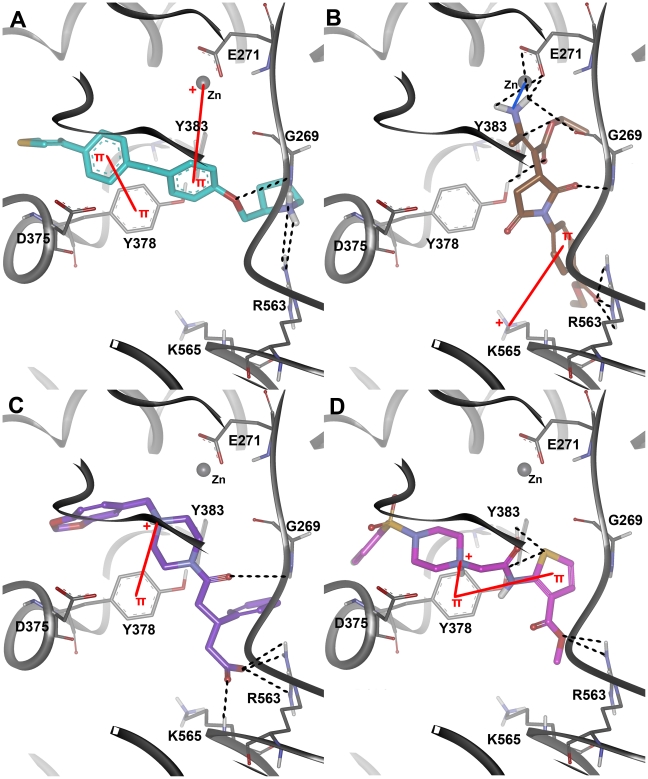
Molecular docking results. The binding modes (A) C15 (B) Hit 1 (C) Hit 2 and (D) Hit 3 at the active site of the enzyme. The amino acid residues and bound ligands are shown in thin and thick stick representations. The hydrogen bond, co-ordinate bond, and π-interactions are shown in green dashed, blue solid and red solid lines.

The binding conformation of the C15 has formed hydrogen bonds with catalytically important R563 and G269 residues through its pyrrolidine nitrogen and oxygen atoms that mapped over the PI and HBD features. The central phenyl ring present in this inhibitor has formed a strong π-cation interaction with divalent metal ion (Zn^2+^) (Figure 11A). Finally 3 hit compounds showing favorable *GOLD_Fitness*, Autodock binding free energy, and highly reactive electronic parameters along with strong molecular interactions were selected and reported as the final inhibitory leads from this study. All of these three final hit compounds were identified through the Pharm-B containing the HBA feature derived from the substrate binding. Five out of seven compounds retrieved through Pharm-A failed to make important molecular interactions and the remaining two compounds (H17 and H18) were rejected based on the Autodock binding free energy (Table 2).

#### Binding mode of Hit 1

**Figure 12:**
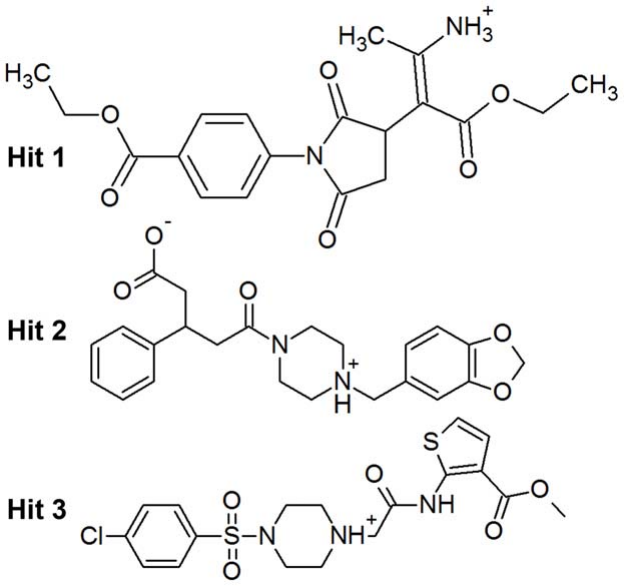
The 2D chemical structures of the final identified hit compounds.

This hit compound has shown a different binding mode compared to that of C15, the most active compound, but bound very tightly with the catalytic components. This hit compound has scored a *GOLD_Fitness* value of 72.669, which is higher than that of C15 but their estimated Autodock binding free energy values were very similar. The molecular interactions observed at the active site included a strong hydrogen bond network and hydrophobic interactions. The hydrogen bond network formed with G269, E271, Y378, Y383, and R563 residues through the ester, oxygen atoms of pyrrolidinone ring, and amino groups present in the compound. A coordinate bond was formed between the metal ion and the primary amino group. The hydrophobic interactions included two π-cation interactions with H299 (not shown in figure) and K565 residues (Figure 11B). This compound is a derivative of pyrrolidinone substituted with two ethyl ester moieties (Figure 12).

#### Binding mode of Hit 2

This compound has bound the active site similar to the highly active inhibitor (C15) and formed hydrogen bond interactions with the same set of residues as observed in C15 complex. The hydrogen bond network included the interactions with G269, R563, and K565 residues through the carboxyl and carbonyl groups present in the compound. Strong hydrophobic interactions including a π-cation interaction were observed with Y267 (not shown) and Y383 residues through the same nitrogen atom of piperazine ring (Figure 11C). This hit compound is a derivative of piperidine with bulky substitutions such as benzodioxol groups (Figure 12). The *GOLD_Fitness* score and Autodock binding free energy values for this compound were 72.383 and −10.04 kcal/mol, respectively. The lower energy gap value was also promising for this compound and increased the reliability to be a potential lead candidate.

#### Binding mode of Hit 3

The molecular interactions exhibited by this hit compound included the hydrogen bond interactions with Y378, Y383, and R563 residues through the ester, sulphur atom of thiophene ring, and amido groups of the compound. Like other hits, Hit 3 has also formed strong hydrophobic interactions such as π-π and π-cation interactions with Y378 (Figure 11D). The *GOLD_Fitness* score and Autodock binding free energy values of this hit compound were 76.419 and −9.94, respectively. This compound is also a derivative of piperazine but with highly different substitutions of thiophene and chlorophenyl groups (Figure 12).

The identified compounds were subjected to novelty study using *SciFinder Scholar* [43] and *PubChem Structure* search tools [44]. This study has confirmed that the identified hits were not reported elsewhere earlier for the inhibition of hLTA4H. These compounds remain virtually identified compounds and the experimental verification of these compounds is required to confirm their inhibitory profiles. The experimental validations of the final hit compounds will be performed in the future using one of the biological assays reported in the literature.

### Conclusions

The hLTA4H enzyme catalyzes hydrolase and aminopeptidase functions by employing its single overlapping binding site. Its hydrolase function converts the epoxy substrate LTA4 to LTB4, a highly potent inflammatory activator of LT cascade. Thus diverse hLTA4H inhibitors were developed by different research groups searching for a most potent anti-inflammatory agent. We selected four structurally similar compounds but diverse in terms of hLTA4H inhibitory profiles to be used in this study aiming at finding the reasons for the difference observed in their inhibitory profiles brought by the small structural difference. The differences in binding modes of the inhibitors along with the hydrogen bond networks and non-bonded interaction energies between the inhibitor and catalytic residues have effectively scripted the differences in the activity. The hydrogen bond network comparison has revealed that the most active compound has formed the hydrogen bond interactions with the positively charged R563 and K565 residues at the active site. These residues were reported to hold the substrate at a particular conformation in such a way that the epoxy group of the substrate binds close to the catalytic machinery and thus the hydrogen bond interaction formed by an inhibitor is considered very essential for inhibitor binding. The structural changes include the secondary structural changes observed at the regions of V306-N308, S379-Y383, and K565 residues that determine the conformation and flexibility of very important catalytic F314, Y378, Y383, and K565 residues. Interestingly, the region of K565 has become more flexible in substrate as well as most active C14 and C15 complexes whereas this region has become rigid by the formation of helix in least active C5 and C13 complexes. The flexibility of F314 residue is different in most active complex (hLTA4H-C15) by forming a helix but not in other complexes. In S379-Y383 region, all of these five residues were present in variable regions but two residues were in variable regions in C14 complex and in least active complexes almost all the residues were involved in helix formation. These observed differences in binding modes, structural changes, and hydrogen bond networks explained the reasons for the potent inhibitory profile of C15, slightly reduced activity of C14, and least active nature of C5 and C13. The dynamic structures of hLTA4H-C15 and hLTA4H-LTA4 complexes were used in the development of two hybrid pharmacophore models and employed in database screening to find potential lead candidates with the pharmacophoric features from both C15 and LTA4. The database hit compounds were further filtered based on the molecular docking results and electronic parameters such as energy gap values. Finally three compounds containing all the identified hybrid features were selected to be used in designing novel and potent hLTA4H inhibitors.
